# From plate to planet: culturally responsive culinary practices for health system innovation

**DOI:** 10.3389/fnut.2024.1476503

**Published:** 2024-10-17

**Authors:** Michelle H. Loy

**Affiliations:** Department of Medicine and Pediatrics, Weill Cornell Medicine, Cornell University, New York, NY, United States

**Keywords:** cultural practices, culturally responsive care, sociocultural health practices, cultural competence, culinary medicine, diversity equity inclusion belonging, environmental sustainability, healthcare system sextuple aims

## Abstract

The field of culinary medicine has gained significant attention for its potential to improve health outcomes through the integration of nutrition and medical practice. However, the cultural dimensions of this interdisciplinary field remain underexplored. Emphasizing the role of sociocultural practices, the paper highlights how culturally appreciative culinary practices can meet the sextuple aim of healthcare system innovation. By examining diverse cultural traditions and their contributions to culinary medicine, this review underscores the importance of culturally attuned approaches in promoting human health. The integration of cultural food wisdom into healthcare practices offers a pathway to more effective and personalized care, stronger patient–provider relationships, diversity/equity/inclusion/belonging, and sustainable food systems.

## 1 Introduction

There is an urgent call for healthcare systems to address the sextuple aims for health improvement, namely (1) quality and experience of patient care; (2) population health outcomes; (3) healthcare provider satisfaction; (4) cost reduction; (5) diversity, equity, inclusion, and belonging (DEIB); and (6) environmental sustainability (1, 2).

Culinary medicine (CM) offers practical approaches to address each of these aims for improved human health. CM has been associated with patient satisfaction (3), improved population health outcomes (4), healthcare provider satisfaction (5), and cost reduction (4) to health systems. Additionally, it offers promise in addressing the two newest aims: diversity, equity inclusion, and belonging (DEIB) (6) and environmental sustainability (2).

Culinary medicine has a long history and has roots in every culture. Discussing food during healthcare visits and offering culturally responsive personalized nutritional dietary plans provide an opportunity to deliver respectful patient care. It honors the diverse food cultures and dietary practices of our patients. Culturally competent healthcare providers and trainees (7) must acknowledge complexities around food and nutrition within different cultural contexts respecting individual unique needs, experiences, preferences, lifestyle, and general wellbeing goals. By embracing culturally attuned, appreciative, and responsive practices, healthcare providers can offer more effective and personalized care. Harnessing culturally relevant models of healthy eating promotes personal and community wellbeing among diverse populations. This review highlights the ways CM can address all six aims, emphasizing the two newest identified aims (DEI and environmental sustainability).

## 2 Culinary medicine and the sextuple aim

Patient care experience and satisfaction, the first aim of healthcare system improvement, is supported by personalized care respecting cultural dietary practices and offering culturally responsive dietary plans. Discussing the diverse food cultures and dietary practices of our patients demonstrates respect and value for indigenous knowledge and traditional food practices. This mutual respect provides a strong base for a collaborative and therapeutic provider–patient relationship (8). Many studies support the role of culturally diverse culinary medicine initiatives in attaining the second aim of healthcare system improvement—improved population health outcomes (9). Clinical parameters (10, 11) (weight, blood glucose, lipids, and HgA1C), food purchasing behavior (12), and culturally relevant health education (13) are some documented effects among Sioux, Latinx, and Asian populations. The third aim, healthcare provider work satisfaction, has been demonstrated to increase among providers engaged in culinary medicine. The enhanced provider–patient relationships through cultural competence and satisfaction from providing personalized and effective care have beneficial downstream effects on healthcare providers, including an increased sense of connection to patients (14–16) of different cultures and generations. Healthcare cost-reducing impacts of culinary medicine (the fourth aim) have been projected for produce prescription programs (17) and other hands-on nutrition programs (18). The use of local and minimally processed foods was associated with household food cost savings (19).

The remainder of this briefing will focus on the implementation of culinary medicine through the lens of diversity, equity, inclusion, and belonging (DEIB) and environmental sustainability, the final two newest aims of the healthcare system reform. Health equity (aim #5) can be promoted through culturally tailored nutrition and addressing social determinants of health related to food access. Environmentally sustainable food practices (aim #6) are rooted in traditional diets, with traditional plant-centric diets producing lower greenhouse gas emissions.

## 3 Cultural diversity in culinary medicine

### 3.1 Nutritional diversity and health benefits

There are well-documented health benefits of nutritionally diverse cultural foods and flavors within cultural heritage eating patterns. Spices, which play a strong role in cultural heritage, have been associated with a plethora of health benefits (20). The bioactive components, polyphenols, and microbiota within traditional foods offer novel mechanistic insight for metabolism and health promotion (21, 22, 121). Advancing cultural diversity education of nutrition educators (23) can harness the benefits of ethnic “superfoods” to optimize healing. Clients can be free to enjoy choosing cost-effective local seasonal produce from ethnic markets rather than work extra shifts to afford the latest marketed “superfood” supplements. Shop with the Doc (24, 25) culinary grocery tours can bring awareness and joy to choosing nutritious, seasonal, culturally revered produce. Diverse traditional cultural diets rely on locally available, minimally processed, nutritious foods rather than processed and packaged foods, which more frequently contain GMO components (26).

Health benefits of spices from various cultural traditions (e.g., TCM, Ayurvedic, Mediterranean, African, and Latin American) have been associated with star anise (27, 28), ajwain, clove (28), cinnamon (28–31), allspice (28, 29), oregano (28, 29), cumin (29) black cumin (30), coriander (29), garlic (29, 32), ginger (29, 30), turmeric (29), caraway (33), parsley (29), black pepper (29), allium (29, 34–36), paprika (29), chili powder (29), rosemary (29), cilantro (29), thyme (29), bayleaf (29), cardamom (29), sage (29), and dillweed (37).

Benefits include blood pressure (29), glucose metabolism (30, 31), reduction in advanced glycation end products (28), cancer risk reduction (34), gut microbiome modulation (38), and immune health (27). Spices confer selective inhibiting effects on pathogenic organisms (Candida, Clostridium, and Bacteroides) while having no effect on beneficial organisms (Lactobacillus and Bifidobacterium) (33). Culinary amounts (2 tsp. cinnamon daily × 4 weeks had effects on 24-h glucose measured by continuous glucose monitoring) (31).

### 3.2 Promoting a healthy relationship with food

Enjoying culturally familiar and revered foods can promote a healthy relationship with food (39). The benefits of introducing culturally diverse foods and spices include less picky eating (40), increased acceptance throughout the lifespan (41), intake of vegetables (42–44), positive emotional context, family bonding, connection, flexibility, and enjoyment. Conversely, the risks of restriction include allergy risk (45, 46) and exacerbation of food anxiety in avoidant restrictive food intake disorder (ARFID) (47). Expanding food variety, increasing pleasure around food, and creating positive emotional context surrounding food are particularly beneficial in situations of food anxiety, ARFID, and disordered eating (which has increased with post-COVID-19 mental stress).

### 3.3 Culturally diverse diets preserving connection, heritage, and health

Elevating and embracing the cultural food wisdom of our patients within the medical visit preserves cultural heritage and supports the idea that there are many “best diets” (48), not just the well-studied Mediterranean Diet. For example, oldways (49) and Blue Zones (50–52) have also been associated with vitality and longevity.

Some foods common in traditional heritage healthy dietary patterns are often less commonly consumed in Western diets. These traditions deserve recognition and support wherever possible. For example, studies suggest that *Saccharomyces boulardii* may play a role in the prevention and treatment of certain gastrointestinal diseases (53–56). This tropical yeast was first isolated from lychee and mangosteen fruit peel (57) by the French scientist Henry Boulard, who was searching for a yeast strain that could withstand heat for wine production. He discovered this “modern probiotic” during his travels to Southeast Asia in the 1920s, after noticing that the natives who drank tea made from Mangosteen skins and other tropical fruits experienced fewer diarrhea symptoms from cholera.

One practical way to recognize and support traditional dietary patterns during medical visits is to ask patients to share their stories about food. Consider inquiring (58) about familiar tastes and foods while growing up, details around taste, smell, feelings, sensations, mood around the table, and memories of preparers. This may open the way for discussion about special cooking (or fermentation) methods, recipes, ingredients, or cultural food events. Not only do cultural and religious food community events preserve heritage, but some also involve philanthropy, commensality, hospitality, and sharing (59–61), thus contributing to equity.

Many cultural food preparation methods and techniques, such as sprouting (25) or fermentation (25, 62) have known health benefits (63). Examples of fermented foods with health benefits include fermented rice (jiu niang) (64, 65), injera (66), dosa (67), natto (68), tempeh (69, 70), sauerkraut (122), yogurt (71), kefir (72), and kombucha (73).

## 4 Addressing socioeconomic diversity in food choices

The role of cultural social determinants of health in acquiring and preparing food for populations with socioeconomic diversity cannot be underestimated. Food choices are often affected by biological, cultural, and societal filters (6, 58, 74, 75).

### 4.1 Special populations

Caregivers within food-insecure household settings face tremendous stress, often working multiple jobs, seeking to avoid wasting food, and may even seek to maximize calories by choosing fast food items with the highest number of calories for the price. Taking into account Satter’s Hierarchy of Food Needs (76), families must first be provided with the resources to meet their immediate needs (77) and then connected to additional resources (78, 79) that allow them to explore a variety of novel foods without the fear of cost burden or waste (74).

These caregivers also include students, many of whom are first-generation parents or caregivers, non-traditional in age, and/or identify as being part of minority groups (80, 81). Sadly, food insecurity among students, including those in postsecondary education, has been on the rise (82–85), with minority and first-generation students facing the highest risk of food insecurity (86, 87). Providing culturally diverse food choices is one way to create a culture at higher institutions supporting food security and health equity.

### 4.2 Inclusive options

Providing inclusive dietary options for special food needs, including medical restrictions, religious dietary restrictions, and food allergies (vegetarian, vegan, halal, kosher, gluten-free, lactose-free, and allergen-friendly) in schools, hospitals, and communities is a baseline requirement, and a variety in cuisine offerings (88) (Japanese, Italian, and Australian) can increase nutritional quality.

Institutions and communities can consider offering alternative ingredients that are cost-effective or more readily available. Within global cuisine, there are diverse options to build basic flavors. For example, salty taste can be imparted through salt, soy sauce, fish sauce, celery, seaweed, and miso, and sourness can be conveyed through apple cider vinegar, rice wine vinegar, citrus, and tamarind.

### 4.3 Ethnic grocery stores

Perhaps “food swamps” should be reframed as “food havens” (75), focusing on the role of local ethnic markets (including dollar bins), bodegas, and farms as underestimated treasure troves of food (89). Patients need to be encouraged to enjoy their “ethnic superfoods” (often found locally and in abundance at ethnic grocery stores) rather than focusing time, money, and resources on acquiring costly “trendy superfoods” (90). Such superfoods are often heavily marketed and processed, stripping them of their nutritional and price value (91).

Providing families with resources for access to healthy foods in low-resource environments, such as week on WIC (78) and “Cooking healthily on a penny” (92–94), can have effects on purchasing behavior and consumption of fresh fruits and vegetables.

### 4.4 Home-cooked meals

The role of rising food costs and inflation have also led to an interest in home-cooked meals. In the Seattle Obesity Study (95), home-cooked dinners were associated with higher Healthy Eating Index scores and reduced *per capita* food expenditures. In contrast, frequent eating out was associated with higher expenditures and lower dietary quality. Food away from home (FAFH) frequency is associated with adverse weight and cardiovascular outcomes (96) and lower overall Healthy Eating Index scores [fewer servings of greens and beans, total and whole fruits; and a higher intake of saturated fats (97) and added sugars (98)]. A greater amount of time spent on home food preparation was associated with a significantly more frequent intake of vegetables, salads, fruits, and fruit juices. Spending <1 h/day on food preparation was associated with significantly more money spent on FAFH and more frequent use of fast-food restaurants than those who spent more time on food preparation (99). Away food contained less dietary fiber, calcium, and iron on a per-calorie basis (97). One study showed variations in socioeconomic and race/ethnicity differences in home dinner preparation habits, indicating that households with foreign-born reference persons and households with dependents tend to cook more dinners at home (100). Regardless of income level, more frequent cooking at home was associated with better diet quality (101). Providing resources for quick, easy, culturally familiar meals at home can have far-reaching public health benefits.

### 4.5 Family meal benefits

Family meals are powerful tools for nurturing physical and emotional health. The frequency of family meals has been associated with improved dietary quality (102), rates of chronic disease, mental (103) and emotional wellbeing (104), and overall positive outcomes (105). Connections to families and cultural values have been shown to be powerful buffers in promoting mental health among minority college students facing stressors (106). Family meals centered around cultural favorites can be especially nourishing.

## 5 Environmental sustainability and culinary medicine

Many traditional cultural diets rely on seasonal (107) locally available, minimally processed, nutritious foods. Many of these foods and preparation methods are plant-centric and low in carbon emissions. These sustainable food practices are relevant not only at the local level (108) but also within the health systems.

### 5.1 Institutions

The use of plant-forward dishes has been associated with lower greenhouse gas emissions in healthcare systems (109) and campuses (110, 111). Emphasizing the procurement of food for local ecosystem also supports sustainable food practices. The export of quinoa is one tale of caution (112, 113).

### 5.2 Upcycling

Many cultures have a tradition of “upcycling” foods by using products that would otherwise go to waste in the food supply chain, yet contain valuable bioactive compounds that confer health benefits (114). Examples might include fruit rinds, vegetable pulps, extracted fibers from plants, or corn silk.

## 6 Clinical applications of culturally responsive culinary medicine

Culturally responsive healthcare through culinary medicine can be applied in a variety of healthcare settings.

### 6.1 Medical education

In recent years, medical schools have started to include justice and advocacy in their curriculum. For example, in one Justice and Advocacy in Medicine course for first-year medical students, culturally relevant cases using traditional Chinese medicine principles to teach nutrition to older Asian adults from the local area highlight cultural competency and health equity applied to patient care (115). A literature review of DEIB in nutrition education provided a recommended checklist for culturally competent nutrition education (7). All healthcare trainees and providers need to be aware of and responsive to the dietary habits and needs of diverse patient populations, diversifying the foods recommended to their clients. Furthermore, increased diversity among professionals with representation of all backgrounds will allow patients to see their cultures embraced (116).

### 6.2 History taking and case examples

Integrating ethnographic food questions into patient history (58) is a helpful tool within patient encounters. Past case studies highlighting successful integration of cultural culinary practices in healthcare settings include using cultural foods to support an oncology patient’s nutrition, making healthy pizza together with an adolescent of Mediterranean origin suffering from a metabolic condition, recommending asafetida to a patient struggling with IBS symptoms, incorporating the five flavors of TCM and 6 flavors of Ayurvedic medicine in expectant mothers’ diet (prenatal palate), and making Hungarian sauerkraut with an older Eastern European patient dealing with chronic pain and lack of purpose.

Adding detailed ethnographic food life questions to standard patient history and clinical encounters provides a broader cultural approach beyond standard food intake questions. It delves into the patient’s emotional, cultural, and familial relationship with food and food choices, for example: asking “what food and flavors take you right back home?,” “what are your food rules?” “how are you learning to care more about food in your life?” (58). These questions often lead to rich discussion that ultimately has lasting effects on patient’s sense of self-efficacy and motivation for behavioral change.

### 6.3 Group visits, community, and employee outreach

Culturally sensitive culinary medicine can be applied to group visits, community outreach, and employee health initiatives. Insurance billable shared medical appointments/group visits (117) extend access to quality care. Group topics may focus on metabolic health, lifestyle change (118, 119), intuitive eating, positive relationships with food, seasonal eating, TCM/ayurvedic dietary patterns (120) healthy detoxification, prenatal health, and oncology (119, 120).

Programs such as Shop with a Doc, farm-to-table events, or cooking demonstrations (121) for employees/staff are other ways to incorporate culturally diverse foods into community outreach education.

## 7 Conclusion

Culinary medicine that emphasizes culturally relevant, nutritionally diverse whole plant foods offers delicious, healthy, practical, and affordable tools to promote sustainable health and wellbeing for patients and clinicians from diverse cultural and socioeconomic backgrounds.

Culturally responsive culinary medicine meets all six aims of healthcare institution reform, including the two newest aims: DEIB and environmental sustainability. Practical tools for integrating cultural food wisdom into medical education and healthcare practices are shared.

Future directions for research and implementation in health systems should include studies that consider culturally relevant topics in the design and implementation of medical education and nutritional studies (8).

## Data Availability

The original contributions presented in the study are included in the article/supplementary material, further inquiries can be directed to the corresponding author.
